# Sexual wellbeing in early adolescence: a cross-sectional assessment among girls and boys in urban Indonesia

**DOI:** 10.1186/s12978-021-01199-4

**Published:** 2021-07-20

**Authors:** Anna E. Kågesten, Anggriyani Wahyu Pinandari, Anna Page, Siswanto Agus Wilopo, Miranda van Reeuwijk

**Affiliations:** 1 Global and Sexual Health, Department of Global Public Health, Karolinska Institutet, Tomtebodavägen 18A, 17177 Stockholm, Sweden; 2 Center for Reproductive Health, Universitas Gadjah Mada, Yogyakarta, Indonesia; 3 Rutgers, Utrecht, Netherlands; 4 Department of Biostatistics, Epidemiology and Population Health, Universitas Gadjah Mada, Yogyakarta, Indonesia

**Keywords:** Sexual wellbeing, Healthy sexuality, Sexual and Reproductive Health and Rights, Early adolescence, Young people, Asia

## Abstract

**Background:**

Early adolescence (ages 10–14) is a critical period of physical, cognitive, social and emotional development, which affect sexual and reproductive health and rights (SRHR). Yet, little is known about positive or healthy aspects of sexuality development during this period of life, especially in South East Asia where sexual norms remain restrictive. The objective of this study is to assess the prevalence and correlates of sexual wellbeing among early adolescent girls and boys ages 10–14 years in Indonesia.

**Methods:**

Data for this cross-sectional study were collected as part of the Global Early Adolescent Study via a school-based survey in three Indonesian urban sites in 2018 (N = 4309). We assessed the prevalence of multiple indicators of sexual wellbeing (e.g. SRHR knowledge and communication, gender attitudes, body satisfaction, self-efficacy, freedom from violence) and tested for differences by sex using Chi-square, Student *t*-test, and Wilcoxon rank-sum test. Multivariable logistic regression models were used to assess the adjusted odds ratio of selected indicators in relation to sociodemographic factors, romantic relationship status, and sexual activities.

**Results:**

The mean age of students was 12 years (53% girls); 90% had started puberty. SRHR knowledge and communication was low overall, but higher among boys than girls. Boys were more likely than girls to report high body satisfaction, less feelings of guilt in relation to sexuality, but also to have experienced physical peer violence. In contrast, girls were more likely to hold gender equal attitudes, greater perceived self-efficacy to say ‘no’, and to report being bullied by boys. In multivariable models, romantic relationship experiences, perceived voice (boys and girls) and decision-making (girls) were associated with three or more indicators of sexual wellbeing.

**Conclusions:**

While young adolescents in Indonesia score high on some aspects of sexual wellbeing, misconceptions, feelings of guilt and uncertainties related to sexuality are common, with clear gender differences. These findings confirm the need for comprehensive sexuality education that begins early in adolescence.

**Supplementary Information:**

The online version contains supplementary material available at 10.1186/s12978-021-01199-4.

## Plain language summary

Ages 10–14 years, known as early adolescence, is a fundamental time when young people become increasingly aware of their bodies, gender and sexuality. Yet, little is known about positive or healthy aspects of sexuality development during this period of life, as most studies tend to focus on risky outcomes such as teenage pregnancy and sexually transmitted infections. In this study, we conducted a survey with primary school students aged 10–14 years in Indonesia, asking them about sexual wellbeing from a broad sense, such as their body image and knowledge about sexual and reproductive health and rights. We analyzed responses from 4309 participants using statistical methods and compared the results between boys and girls. We found that misconceptions, feelings of guilt and lack of knowledge related to sexual and reproductive health were common, with clear differences between boys and girls. Our study provides new information about healthy sexuality development in an Asian setting where young people’s sexual and reproductive health is often considered taboo. The results can be used to guide programs and research to improve sexual and reproductive health and rights in Indonesia and other low-income settings. In particular, comprehensive sexuality education may play an important role to support sexual wellbeing by providing factual and age-appropriate information as young people grow up.

**Supplementary Information:**

The online version contains supplementary material available at 10.1186/s12978-021-01199-4.

## Background

In 1994 the International Conference on Population and Development first emphasized the need for countries and governments to invest in adolescent sexual and reproductive health and rights (SRHR) [1]. Since then, the world has seen vast improvements in adolescent SRHR, with significant reductions in teenage pregnancy, child marriage, and female genital mutilation [2] and increased investments in programs and policies to support adolescents’ needs. However, challenges persist, as progress has been patchy across and within countries [2], and adolescent sexuality remains highly stigmatized in many contexts [3].

While addressing these challenges requires a comprehensive approach to SRHR that fully recognizes adolescents’ right to their own bodies and to understand their sexuality, research and programs remain almost exclusively focused on risk reduction and negative consequences such as HIV and unintended pregnancy [2]. In contrast, research on positive aspects of adolescent sexuality (e.g., self-esteem, gender attitudes and consensual relationships) is largely missing from a global perspective [4–8].

The lack of a positive approach becomes problematic given that sexuality development is a universal, multidimensional process that begins long before a young person has had any sexual interactions [4–7]. Healthy adolescent sexuality development is much more than freedom from disease—it is about building social, emotional and cognitive skills that allow young people to achieve a sense of wellbeing in relation to their bodies, their sexuality and relationships [5]. The formation of such skills is especially critical during early adolescence (ages 10–14 years), which is a period of rapid and interconnected developmental changes that lay the foundation for future health and behaviors [9].

While a small but growing field has attempted to explore different aspects of adolescent sexual wellbeing, most of what we know comes from studies in high-income countries with older youth. Using varying conceptualizations, studies have assessed outcomes such as adolescent’s sexual self-concept [10, 11], sexual self-efficacy [10–12], sexual communication [13], sexual attitudes [12, 13], and SRHR knowledge [11, 13]. Research in low- and middle-income countries is scant, with a few examples predominantly from sub-Saharan Africa, such as a cross-sectional assessment of gender attitudes, self-esteem and body image among 10–14-year-olds in Uganda [14].

In this paper, we focus on the emerging sexual wellbeing of young adolescents in Indonesia, a setting where sexual norms remain restrictive [15]. Nonetheless, evidence indicates that young people in contemporary Indonesia want to, and are, exploring their own sexuality and intimate relationships before marriage. According to the 2018 Indonesian Demographic and Health Survey [16], 98–99% of never-married 15–19-year-olds say that women should remain virgins at marriage, and only 0.9% of never-married girls and 3.6% of boys report having had sexual intercourse. Meanwhile, 75% of girls and 78% of boys had dated someone, with a third starting during early adolescence; and of those who ever dated, up to 56% of girls and 66% of boys report non-coital experiences (petting, kissing or holding hands) [16]. A recent qualitative online study with 16–24-year-olds from urban Indonesian communities further revealed complex patterns by which young people are actively learning about sexuality and how to take care of themselves, while simultaneously embracing dominant (restrictive) norms [15].

Using data collected as part of the Global Early Adolescent Study, our paper has two aims: (1) to assess the prevalence of a broad range of sexual wellbeing indicators among 10–14-year-old girls and boys residing in three urban regions of Indonesia; and (2) to investigate the association between selected sexual wellbeing indicators with sociodemographic factors as well as romantic relationship status and sexual activities.

### Conceptual framework

In the current study, we used the conceptual framework for adolescent sexual wellbeing developed by Kågesten and van Reeuwijk [17]. The framework highlights six interrelated domains of competencies—in the form of knowledge, attitudes and skills—that are central for healthy sexuality development in adolescence: sexual literacy, gender equal attitudes, respect for human rights and understanding consent, critical reflection skills, coping skills, and interpersonal skills. Together, these competencies have the potential to impede or enhance adolescents’ sense of sexual wellbeing both in relation to themselves (e.g. body satisfaction, self-efficacy) and others (e.g. consensual social or sexual interactions). In the current study, we focused on these outcomes to explore gender differences in prevalence and correlates, and use the terms sexual wellbeing and healthy sexuality interchangeably.

## Methods

### Study design and context

We conducted a cross-sectional study as part of the baseline Indonesian arm of the longitudinal Global Early Adolescent Study (GEAS, www.geastudy.org). In Indonesia, the GEAS is part of the *Explore4Action* quasi-experimental trial evaluating the effect of the comprehensive sexuality education intervention *SETARA* for high-school students (taught in the 7th and 8th grade) on adolescent SRHR. The project is a collaboration between Rutgers, University Gadjah Mada and the Indonesia Planned Parenthood Association, with support from Johns Hopkins University and Karolinska Institutet. Baseline data were collected in 2018 at intervention and control schools across three sites: Bandar Lampung (Sumatra), Semarang (Java) and Denpasar (Bali), with two follow-ups planned for 2021 and 2022. The sites are predominately urban with different ethnic, cultural, religious and social-economic characteristics. Bandar Lampung is a multi-ethnic, Muslim majority city characterized by farming, forestry, mining, fishing, industry and retail. It is generally more Islam conservative than Semarang, which is a Muslim and Javanese majority city predominated by retail, construction and other industries. In contrast, Denpasar is mainly Balinese with Hinduism as main religion, and tourism dominating the economy [18].

The study received ethical approval from the Medical Research and Ethics Committee at the Faculty of Medicine, Public Health and Nursing, University Gadjah Mada, and the Johns Hopkins Bloomberg School of Public Health Institutional Review Board for secondary data analysis. We used the STROBE cross-sectional reporting guidelines to guide the development of the current manuscript [19].

### Participants and sampling

To be included in this baseline survey, participants had to be aged 10–14 years as per the Global Early Adolescent Study inclusion criteria, in grade 7 (starting point for the SETARA intervention) at one of the 18 selected schools, and provide written parental/guardian consent as well as their own assent to participate. In each site, three schools were assigned to the intervention, and matched with three nearby control schools with similar characteristics. In total, 4684 boys and girls participated in the baseline survey: 1414 in Bandar Lampung, 1517 in Semarang, and 1753 in Denpasar (response rate 75.7%, 92.8% and 99%, respectively) [18].

Of the total sample, 2550 participants had complete data for all 18 sexual wellbeing outcome variables assessed as part of the current study, meaning 46.5% of participants were missing one or more variables, mainly due to “don’t know” and “refuse to answer” responses. Most were missing 1 (21.3%) or 2–3 (12.6%) outcomes, wherefore we coded don’t know/refuse answers as “no” (0) to retain as much of the sample as possible. To assure against bias due to recoding, we conducted rigorous sensitivity analyses to compare the distribution in outcomes and covariates when restricting the sample to those with complete data on all outcomes included in our analysis (N = 2550) versus coding don’t know/refuse as no (N = 4309). The sensitivity analysis (available upon request) did not indicate any systematic differences in the sample characteristics, with the exception that everyone (100%) in the complete case had started puberty (compared to 92.8% in the full sample) and scored higher on a couple of outcomes (for example, a higher proportion of those with no missing values reported ever talking about SRHR with someone else). This indicated that participants in the complete case sample might have been more experienced and comfortable responding to SRHR questions. Nonetheless, results from bivariate and multivariate analyses were similar for the two approaches in the sensitivity analysis. We therefore proceeded with the larger sample of N = 4309.

### Procedures

Data for the baseline survey were collected at the schools between August and October 2018 by a team of trained data collectors using computer-assisted self-interviewing via tablets, and computer-assisted personal interviewing for adolescents with low literacy levels. The standardized survey covered sociodemographics, family, peer, school and neighborhood factors, gender attitudes, physical and mental health, puberty, empowerment, violence, romantic relationships, and sexual and reproductive health. Data were uploaded to a secure server at Universitas Gadjah Mada with regular quality checks. A formal protocol ensured child protection and reporting of child abuse.

### Measures

We included multiple outcome variables across different domains of sexual wellbeing, guided by the conceptual framework. Below we provide a brief overview of the outcome variables; a detailed description is available in Table 1.

**Table 1:** Definitions and measures of sexual wellbeing outcome variables

Variable by domain	Survey question(s) and response options	Measurement in analysis
*Sexual literacy and communication*		
Knowledge about pregnancy	∙ A girl can get pregnant the first time that she has sexual intercourse ∙ A girl can get pregnant after kissing or hugging ∙ A girl can swallow a pill every day to protect against pregnancy ∙ Using a condom can protect against pregnancy ∙ A girl can have a shot or injection that will protect against pregnancy ∙ A girl can use herbs to prevent a pregnancy *(yes, no, don’t know)*	Continuous, mean score of correct answers (0–6)
Knowledge about HIV	∙ A boy/girl can get HIV the first time that he/she has sexual intercourse ∙ Using a condom can protect against HIV ∙ You can get HIV through kissing ∙ A girl or boy can swallow a pill before having sex that will protect against HIV *(yes, no, don’t know)*	Continuous, mean score of correct answers (0–4)
SRHR knowledge	Combining questions about pregnancy and HIV	Continuous, mean total score (0–10) Dichotomized into: Low ≤ 50% correct High > 50% correct
SRHR communication	∙ Have you talked with anyone about the body changes that happen as boys and girls grow up? ∙ Have you ever discussed the following topics with anyone? Sexual relationships Pregnancy and how it happens Contraception *(yes, no, don’t know)*	Categorical 1 = talked about SRHR (puberty, sexual relationships, pregnancy, contraception) with someone 0 = did not talk 2 = don’t know
SRHR topics ever discussed (only among those who reported ever talking about SRHR)	∙ Body changes/puberty ∙ Sexual relations ∙ Pregnancy ∙ Contraceptives	Categorical 1 = talked about this topic with someone 0 = did not talk 2 = don’t know
*Gender attitudes*		
Sexual double standard (SDS)	∙ Boys have girlfriends to show off to their friends ∙ Adolescent boys fool girls into having sex ∙ Boys tell girls that they love them when they don’t ∙ Adolescent boys loose interest in a girl after they have sex with her ∙ Adolescent girls should avoid boys because they trick them into having sex ∙ Girls are the victims of rumors if they have boyfriends *(1* = *strongly agree, 2* = *agree, 3* = *neither agree nor disagree, 4* = *disagree, 5* = *strongly disagree)*	Continuous, 6-item scale with composite score (mean) 1–5; higher scores = higher agreement Cronbach’s alpha = 0.8 Dichotomized at the median into agree vs disagree
Gender stereotypical traits (GST)	∙ Boys should always defend themselves even if it means fighting ∙ It’s important for boys to show they are tough even if they are nervous inside ∙ Boys who behave like girls are considered weak ∙ Boys should be able to show their feelings without fear of being teased ∙ Boys should be raised to be tough so can overcome any difficulties in life ∙ A boy should always have the final say about decision with his girlfriend ∙ Girls are expected to be humble ∙ Girls should avoid raising their voice to be lady like ∙ Girls need their parents’ protection more than boys *(1* = *strongly agree, 2* = *agree, 3* = *neither agree nor disagree, 4* = *disagree, 5* = *strongly disagree)*	Continuous, 7-item scale with composite score (mean) 1–5 Cronbach’s alpha = 0.72 Dichotomized at the median into agree vs disagree
Gender stereotypical roles (GSR)	∙ A woman’s role is taking care of her home and family. (GN39) ∙ A man should have the final word about decisions in the home. (GN40) ∙ Boys and girls should be equally responsible for household chores. (GN38) ∙ A woman should obey her husband in all matters. (GN41) ∙ Men should be the ones who bring money home for the family, not women. (GN44) *(1* = *strongly agree, 2* = *agree, 3* = *neither agree nor disagree, 4* = *disagree, 5* = *strongly disagree)*	Continuous, 5-item scale with composite score (mean) 1–5 Cronbach’s alpha = 0.77 Dichotomized at the median into agree vs disagree
Attitudes towards gender-related teasing	∙ It is okay to tease a girl who acts like a boy ∙ It is okay to tease a boy who acts like a girl *(1* = *strongly agree, 2* = *agree, 3* = *neither agree nor disagree, 4* = *disagree, 5* = *strongly disagree)*	Dichotomous 0 = disagree/neutral 1 = agree
*Comfort with body and emerging sexuality*		
Body satisfaction	∙ On the whole, I am satisfied with my body ∙ I like the way I look ∙ I like looking at my body ∙ I feel like I am beautiful/handsome *(1* = *strongly agree, 2* = *agree, 3* = *neither agree nor disagree, 4* = *disagree, 5* = *strongly disagree)*	Continuous, 4-item scale with composite score (mean) 1–5 Cronbach’s alpha = 0.72 Dichotomous 0 = neg/neutral (≤ 3) 1 = positive (> 3)
Comfort with pubertal development	∙ I like the fact that I am becoming a man (boys) ∙ I like the fact that I am becoming a woman (girls) *(1* = *strongly agree, 2* = *agree, 3* = *neither agree nor disagree, 4* = *disagree, 5* = *strongly disagree)*	Categorical 0 = disagree 1 = neutral/neither 2 = agree
Feelings of guilt about sexuality	∙ Looking at myself naked when I am alone I would feel… ∙ If I were romantically attracted to someone else I would feel… ∙ If I were to touch the private parts of my body I would feel… ∙ If I had sexual feelings I would feel… *(1* = *not guilty at all, 2* = *not very guilty, 3* = *a little guilty, 4* = *somewhat guilty, 5* = *very guilty)*	Continuous, 4-item scale with composite score (mean) ranging from 1–5 Cronbach’s alpha = 0.65 Dichotomous 0 = low (< 3) 1 = high (≥ 3)
Normal to be curious about love/sexuality	∙ It is normal for adolescents to be curious about love and sexuality *(1* = *not true at all, 2* = *not very true, 3* = *somewhat true, 4* = *very true, don’t know)*	Categorical 0 = no (1–2) 1 = yes (3–4) 2 = don’t know
*Perceived relational self-efficacy*		
Communicate romantic feelings	How confident do you think you would be… ∙ Telling a boy or girl that you like them *(1* = *not at all confident, 2* = *not very confident, 3* = *a little confident, 4* = *somewhat confident, 5* = *very confident)*	Dichotomous 0 = low (1–2) 1 = high (3–5)
Say “no” to unwanted interaction	How confident do you think you would be… ∙ Telling a boy or girl no if they were doing something that you don’t want *(1* = *not at all confident, 2* = *not very confident, 3* = *a little confident, 4* = *somewhat confident, 5* = *very confident)*	Dichotomous 0 = low (1–2) 1 = high (3–5)
Prevent pregnancy	How confident do you think you would be… ∙ Talking to a boyfriend or girlfriend about contraception ∙ Obtaining information on prevention of pregnancy ∙ Getting contraception if you need it *(1* = *not at all confident, 2* = *not very confident, 3* = *a little confident, 4* = *somewhat confident, 5* = *very confident)*	Continuous, 3-item scale with composite score (mean) ranging from 1–5 Cronbach’s alpha = 0.81 Categorical 0 = low (1–2) 1 = high (3–5)
*Freedom from peer bullying and violence*		
Bullied by peers during last 6 months	∙ During the last six months, have you been teased or called names by someone? *(yes, by a girl; yes, by a boy; yes, by both boys and girls, don’t know)*	Categorical based on sex of perpetrator 0 = no 1 = yes, both boys and girls 2 = yes, opposite sex only
Bullying due to gender last 6 months (only among those bullied)	∙ If you were teased or called names, do you think this was because… You are a girl You are a boy The person thought you were acting like a girl The person thought you were acting like a boy *(yes, no, don’t know)*	Combined bullying due to being boy/girl with acting like opposite sex into “gender-related reason” 0 = other reason 1 = due to gender
Physical violence victimization by peers during last 6 months	∙ During the last 6 months, have you ever been slapped, hit or otherwise physically hurt by a boy or girl in a way that you did not want? *(yes, by a girl; yes, by a boy; yes, by both boys and girls, don’t know)*	Categorical based on perpetrator sex 0 = no 1 = yes, both boys and girls 2 = yes, opposite sex only

#### Sexual literacy and communication

HIV and pregnancy knowledge were assessed by aggregating the mean score of 10 correct responses, further dichotomized into low (< 50% correct) and high (≥ 50% correct) SRHR knowledge. SRHR communication was assessed by combining five items related to ever having discussed four topics with someone else: body changes, sexual relationships, pregnancy and/or contraceptives.

#### Gender attitudes

Three scales developed and validated as part of the GEAS [20] were used to assess perceptions about gender norms, including: (1) sexual double standards, measuring expectations for boys and girls in sexual relationships (SDS); (2) gender stereotypical traits (GST), reflecting traditionally masculine vs. feminine characteristics; and (3) gender stereotypical roles (GSR), tapping into expectations about the roles and power of boys and girls. Response options ranged from 1 to 5 and were averaged into mean scores (higher = more gender equal attitudes) and dichotomized at the median. We also included a single item measuring agreement with gender-related teasing (e.g., it is ok to tease a boy who ‘acts like a girl’).

#### Comfort with body and emerging sexuality

Body satisfaction was assessed via a 4-item, 5-point scale [21], with mean scale scores dichotomized into low (≤ 3) vs. high (> 3). Pubertal comfort was measured via the item “I like the fact that I am becoming a man/woman” (disagree, neutral, agree), and feelings of guilt in relation to sexuality was assessed using a 3-item, 5-point scale measuring perceived guiltiness for looking at oneself naked, being attracted to someone else, having sexual feelings, and/or touching own private body parts (Cronbach’s alpha = 0.65), with scores dichotomized at the median into low (< 3) vs. high (≥ 3) perceived guilt. A single item measured agreement with whether it is normal for adolescents to be curious about love and sexuality.

#### Relational self-efficacy

Two 5-point items were used to measure perceived self-efficacy to communicate romantic feelings vs. consent; and a 3-item scale assessed self-efficacy to prevent pregnancy (Cronbach’s alpha = 0.81), with mean scores dichotomized into high (3–5) vs. low (1–2).

#### Freedom from bullying and violence

In early adolescence, sexual wellbeing also includes forming healthy, non-violent social relationships with peers. Bullying by peers in the last 6 months was assessed using two items and categorized into no bullying, bullying by both boys/girls, and bullying by the opposite sex only. The same approach was used for physical peer violence victimization in the last 6 months. For participants reporting to have been bullied, a dichotomous variable measured whether they thought that this was due to gender or not (because they are a boy/girl, or because they ‘acted like’ the opposite sex).

#### Social-ecological covariates

Beyond study site (Denpasar, Semarang, Bandar Lampung), covariates were self-reported at different levels of the social-ecological framework. Participants sex (boy, girl) was measured using the question “are you a boy or a girl?”; for purposes of simplicity we use the terms sex and gender interchangeably. Other individual variables included age, pubertal onset, religion, religiosity, and perceived agency, assessed using two validated 5-point scales [22] to measure (express opinions and be heard) and decision-making (make autonomous choices in daily life). Three variables assessed adolescent’s romantic relationship status and lifetime experiences of non-coital sexual activities (e.g. hugging or kissing with someone “as more than just friends”) as well as penile-vaginal sexual intercourse. Family variables encompassed main caregiver, household structure and perceived parental connectedness. School/community factors included educational aspirations, number of school days missed last month, whether the adolescent ever felt threatened at school, and access to social media. A detailed description of all covariates and their measures is available in Additional file 1: Appendix Table 1.

### Statistical analysis

We began with exploratory data analysis to assess the distribution in outcomes and covariates and evaluate patterns of missingness. Following the coding of missing values for outcomes (see Participants and sampling), missing values on covariates were imputed using K-nearest neighbor (kNN). Imputation were done separately for the analytical sample (N = 4309) and for the complete case sample (N = 2550), with sensitivity analysis indicating no major differences in the distribution of covariates. We then conducted bivariate analysis using Chi-square, Student *t*-test, and Wilcoxon rank-sum to compare the prevalence of sexual wellbeing outcomes by sex. We also used radar charts to display the distribution of positive outcome responses (i.e., indicating sexual wellbeing) to visualize differences between boys and girls. Next, we selected one outcome variable from each sexual wellbeing domain: SRHR communication; SDS attitudes; body satisfaction; self-efficacy to say no; and freedom from bullying and violence, for bivariate analysis in relation to covariates. These variables were selected based on their relevance to the age group, variation in responses and validity. A series of multivariable logistic regression models estimating adjusted odds ratios (aOR) and associated 95% confidence intervals (CI) were fitted for each of the five outcomes separately for boys vs. girls at the alpha = 0.05 significance level. Backward selection and Akaike’s Information Criterion (AIC) were used to examine the independent contribution of covariates to each of the models, with lower AIC values indicating better fit, and Pearson’s goodness of fit test used to examine relative model fit. The variance inflation factor (VIF) ranged between 1 and 1.20, indicating no multicollinearity in covariates. All analyses were conducted using STATA version 15.1 (StataCorp. 2017. Stata Statistical Software: Release 15. College Station, TX).

## Results

Of the 4309 participants included in the analysis, 47.0% were boys and 53.0% girls with mean age 12.2 years (Table 2). Nine in ten (90.4% boys, 94.9% girls) had started puberty. Islam was the main religion (63.2%) followed by Hinduism (34.3%), reflecting the characteristics of the study sites, with the majority of participants reporting high religiosity—especially among boys (87.8% vs. 83.8% among girls). Perceived agency varied by sex, with girls being more likely than boys to perceive high voice (48.3% vs. 36.8%, p < 0.001) and decision-making (35.8% vs. 32.8%, p = 0.039). Most participants reported no romantic or sexual experiences, although boys were more likely than girls to report ever being in a romantic relationship (45.0% vs. 35.8%, p < 0.001) and having non-coital experiences (31.9% vs. 25.1%, p < 0.001). Boys were also more likely to report having had sexual intercourse, though this was very uncommon and only reported by 2% (N = 49) of boys and 0.2% (N = 5) of girls. In terms of family characteristics, almost nine in ten participants (85.7%) reported living with both parents, with mothers being the main caregiver (88.9%). Girls were more likely than boys to report high parental connectedness (66.6% vs. 62%, p = 0.007), to aspire to complete university (84.9% vs. 68.4%, p < 0.001) and to have missed no school days in the last month (73.5% vs. 67.1%, p < 0.001). Girls were also less likely than boys to ever have felt threatened at school (15.2% vs. 19.9%, p < 0.001). Almost all (93.2%) of the total sample reported having access to a social media account.

**Table 2:** Sample characteristics (N = 4309)

Variable	Total	Boys	Girls	*p*
	N = 4309	N = 2031	N = 2278	
Site				0.14
Bandar Lampung	1143 (26.5%)	543 (26.7%)	600 (26.3%)	
Denpasar	1682 (39.0%)	818 (40.3%)	864 (37.9%)	
Semarang	1484 (34.4%)	670 (33.0%)	814 (35.7%)	
*Individual*				
Age (mean)	12.2 (0.5)	12.2 (0.6)	12.1 (0.5)	< 0.001
10–12 years	3356 (77.9%)	1499 (73.8%)	1857 (81.5%)	
13–14 years	953 (22.1%)	532 (26.2%)	421 (18.5%)	
Pubertal onset				< 0.001
Prepubertal	310 (7.2%)	194 (9.6%)	116 (5.1%)	
Pubertal	3999 (92.8%)	1837 (90.4%)	2162 (94.9%)	
Religion				0.55
Islam	2725 (63.2%)	1264 (62.2%)	1461 (64.1%)	
Hinduism	1477 (34.3%)	715 (35.2%)	762 (33.5%)	
Christian/other	91 (2.5%)	43 (2.5%)	48 (2.4%)	
Religiosity				< 0.001
Low	618 (14.3%)	248 (12.2%)	370 (16.2%)	
High	3691 (85.7%)	1783 (87.8%)	1908 (83.8%)	
Perceived voice (median, IQR)	3.0 (2.6, 3.4)	2.9 (2.3, 3.3)	3.0 (2.7, 3.4)	< 0.001
Low	2461 (57.1%)	1284 (63.2%)	1177 (51.7%)	< 0.001
High	1848 (42.9%)	747 (36.8%)	1101 (48.3%)	
Decision-making (median, IQR)	3.0 (2.5, 3.2)	3.0 (2.3, 3.2)	3.0 (2.5, 3.2)	0.003
Low	2828 (65.6%)	1365 (67.2%)	1463 (64.2%)	0.039
High	1481 (34.4%)	666 (32.8%)	815 (35.8%)	
Relationship status				< 0.001
Never in relationship	2581 (59.9%)	1118 (55.0%)	1463 (64.2%)	
Ever but not currently	994 (23.1%)	445 (21.9%)	549 (24.1%)	
Currently in relationship	734 (17.0%)	468 (23.0%)	266 (11.7%)	
Ever non-coital sexual activities				< 0.001
No	3089 (71.7%)	1383 (68.1%)	1706 (74.9%)	
Yes	1220 (28.3%)	648 (31.9%)	572 (25.1%)	
Ever sexual intercourse				< 0.001
No	4255 (98.7%)	1982 (97.6%)	2273 (99.8%)	
Yes	54 (1.3%)	49 (2.4%)	5 (0.2%)	
*Family*				
Main caregiver				0.027
Mother	3830 (88.9%)	1772 (87.2%)	2058 (90.3%)	
Father	240 (5.6%)	129 (6.4%)	111 (4.9%)	
Sibling	21 (0.5%)	12 (0.6%)	9 (0.4%)	
Grandparent	119 (2.8%)	67 (3.3%)	52 (2.3%)	
Other	99 (2.3%)	51 (2.5%)	48 (2.1%)	
Living with				< 0.001
Both parents	3695 (85.7%)	1717 (84.5%)	1978 (86.8%)	
One parent	386 (9.0%)	171 (8.4%)	215 (9.4%)	
Grandparents/other	228 (5.3%)	143 (7.0%)	85 (3.7%)	
Parental connectedness				0.007
Low	507 (11.8%)	254 (12.5%)	253 (11.1%)	
Somewhat	1024 (23.8%)	517 (25.5%)	507 (22.3%)	
High	2778 (64.5%)	1260 (62.0%)	1518 (66.6%)	
*School/community*				
Educational aspirations				< 0.001
≤ Junior high	128 (3.0%)	88 (4.3%)	40 (1.8%)	
Senior high/dipl	784 (18.2%)	513 (25.3%)	271 (11.9%)	
University	3322 (77.1%)	1389 (68.4%)	1933 (84.9%)	
Other	75 (1.7%)	41 (2.0%)	34 (1.5%)	
School days missed last month				< 0.001
None	3038 (70.5%)	1363 (67.1%)	1675 (73.5%)	
1–2 days	1114 (25.9%)	580 (28.6%)	534 (23.4%)	
3 or more days	157 (3.6%)	88 (4.3%)	69 (3.0%)	
Ever felt threatened at school				< 0.001
No	3558 (82.6%)	1627 (80.1%)	1931 (84.8%)	< 0.001
Yes	751 (17.4%)	404 (19.9%)	347 (15.2%)	
Access to social media				
No	295 (6.8%)	161 (7.9%)	134 (5.9%)	< 0.01
Yes	4014 (93.2)	1870 (92.1%)	2144 (94.1%)	

### Prevalence of sexual wellbeing

**Fig. 1:**
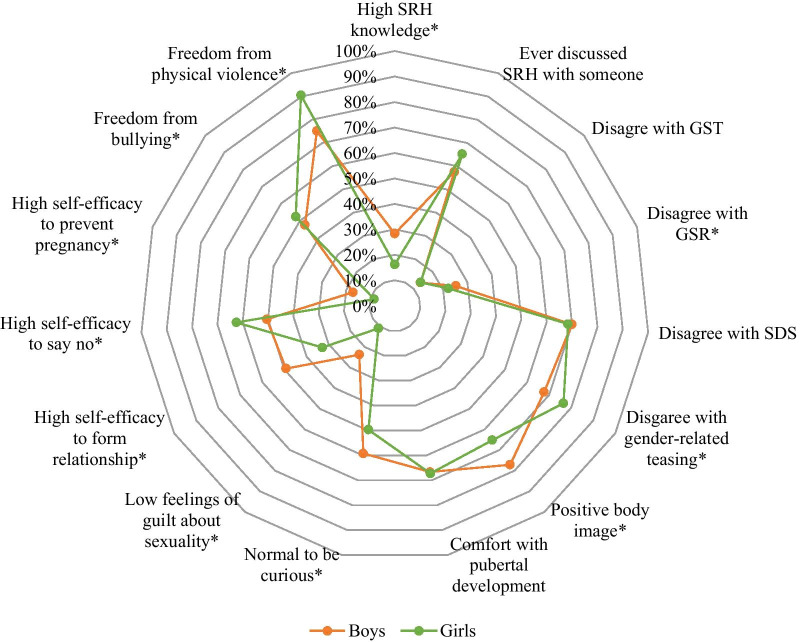
Prevalence of sexual wellbeing indicators among boys and girls in three Indonesian sites. *p < 0.05 comparing the prevalence of sexual wellbeing indicators between boys and girls

Table 3 shows the distribution of the sexual wellbeing indicators by sex and Fig. 1 further visualizes patterns with focus on positive responses. Knowledge about HIV and pregnancy was low overall (mean score 2.6 out of 10 correct answers for the combined questions), but higher for boys where 28.3% had high SRHR knowledge compared to 16.2% for girls (p < 0.001). Two in three boys and girls reported to ever talking with someone else about one or more SRH topics; and among those who ever did so, girls were more likely than boys to have talked about body changes that come with puberty (79.1% vs 69.%, p < 0.001), whereas communication about pregnancy, sexual relationships, and contraceptives were more common for boys (41.6%, 42.4%, 29.3% among boys vs. 19.7%, 34.7%, 15.5% among girls, p < 0.001).

**Table 3:** Prevalence of sexual wellbeing indicators for boys and girls in Indonesia

Variable	Category	Total (N = 4309)	Boys (N = 2031)	Girls (N = 2278)	*p*
		n (%) mean (SD)	n (%) mean (SD)	n (%) mean (SD)	
*Sexual literacy and communication*					
Knowledge about HIV	Mean (SD) range 1–4	0.9 (1.1)	1.1 (1.1)	0.7 (1.0)	< 0.001
Knowledge about pregnancy and contraceptives	Mean (SD) range 1–6	1.7 (1.5)	2.0 (1.6)	1.5 (1.4)	< 0.001
Overall SRHR knowledge	Mean (SD) range 1–10	2.6 (2.3)	3.1 (2.4)	2.2 (2.2)	< 0.001
	Low (< 50% correct)	3364 (78.1%)	1456 (71.7%)	1908 (83.8%)	< 0.001
	High (≥ 50% correct)	945 (21.9%)	575 (28.3%)	370 (16.2%)	
SRHR communication	No	1371 (31.8%)	633 (31.2%)	738 (31.8%)	< 0.387
	Yes	2938 (68.2%)	1398 (68.8%)	1540 (67.6%)	
SRHR topics ever discussed*	Body changes/puberty	2182 (74.3%)	964 (69.0%)	1218 (79.1%)	< 0.001
	Sexual relations	884 (30.1%)	581 (41.6%)	303 (19.7%)	< 0.001
	Pregnancy	1127 (38.4%)	593 (42.4%)	534 (34.7%)	< 0.001
	Contraceptives	649 (22.1%)	410 (29.3%)	239 (15.5%)	< 0.001
*Gender attitudes*					
Sexual double standard	Mean (SD) range 1–5	2.7 (1.0)	2.7 (1.0)	2.7 (1.0)	0.92
Gender-stereotypical traits	Mean (SD) range 1–5	3.9 (0.7)	3.9 (0.7)	3.8 (0.7)	< 0.001
Gender-stereotypical roles	Mean (SD) range 1–5	3.9 (1.0)	3.9 (1.0)	3.8 (0.9)	0.054
Ok to tease a boy/girl who behaves like opposite gender	Do not agree	3118 (72.4%)	1375 (67.7%)	1743 (76.5%)	< 0.001
	Agree	1191 (27.6%)	656 (32.3%)	535 (23.5%)	
*Comfort with body and emerging sexuality*					
Body satisfaction	Mean (SD) range 1–5	3.6 (0.8)	3.8 (0.8)	3.5 (0.8)	< 0.001
	Negative	1258 (29.2%)	466 (22.9%)	792 (34.8%)	< 0.001
	Positive	3051 (70.8%)	1565 (77.1%)	1486 (65.2%)	
Comfort with pubertal development	Do not agree	760 (17.6%)	455 (22.4%)	305 (13.4%)	< 0.001
	Neutral	661 (15.3%)	221 (10.9%)	440 (19.3%)	
	Agree	2888 (67.0%)	1355 (66.7%)	1533 (67.3%)	
Feelings of guilt about sexuality	Low	731 (17.0%)	482 (23.7%)	249 (10.9%)	< 0.001
	High	3578 (83.0%)	1549 (76.3%)	2029 (89.1%)	
Normal to be curious about love/sexuality	Do not agree	1220 (28.3%)	487 (24.0%)	733 (32.2%)	< 0.001
	Agree	2337 (54.2%)	1204 (59.3%)	1133 (49.7%)	
	Do not know	752 (17.5%)	340 (16.7%)	412 (18.1%)	
*Perceived relational self-efficacy*					
Communicate romantic feelings	Low	2559 (59.4%)	1029 (50.7%)	1530 (67.2%)	< 0.001
	High	1750 (40.6%)	1002 (49.3%)	748 (32.8%)	
Say “no” to unwanted interaction	Low	1861 (43.2%)	1006 (49.5%)	855 (37.5%)	< 0.001
	High	2448 (56.8%)	1025 (50.5%)	1423 (62.5%)	
Prevent pregnancy	Low	3767 (87.4%)	1682 (82.8%)	2085 (91.5%)	< 0.001
	High	542 (12.6%)	349 (17.2%)	193 (8.5%)	
*Freedom from peer bullying and violence*					
Bullied by peers in last 6 months	No	2155 (50.0%)	963 (47.4%)	1192 (52.3%)	< 0.001
	Yes, by both boys/girls	1721 (39.9%)	960 (47.3%)	761 (33.4%)	
	Yes, by opposite sex only	433 (10.0%)	108 (5.3%)	325 (14.3%)	
Reason for bullying^±^	Due to gender	881 (40.9%)	455 (42.6%)	426 (39.2%)	0.111
	Other reason	1273 (59.1%)	613 (57.4%)	660 (60.8%)	
Physical violence from peers in last 6 months	No	3587 (83.2%)	1525 (75.1%)	2062 (90.5%)	< 0.001
	Yes, by both boys/girls	510 (11.8%)	376 (18.5%)	134 (5.9%)	
	Yes, by opposite sex only	212 (4.9%)	130 (6.4%)	82 (3.6%)	
Any peer violence (combined bullying or physical)	No	882 (43.4%)	1174 (51.5%)	2056 (47.7%)	< 0.001
	Yes	1149 (56.6%)	1104 (48.5%)	2252 (52.3%)	

*Among those who ever discussed SRHR (N = 2938 total, N = 1398 boys, N = 1540 girls)

^±^Among those reporting bullying in the past 6 months (N = 2154 total, N = 1068 boys, N = 1086)

In terms of gender attitudes, both boys and girls had relatively low agreement with sexual double standard norms (mean 2.7 out of 5), but higher for gender-stereotypical roles and traits (mean 3.9)—especially among boys, whereas girls were more likely than boys to disagree that it is ok to tease a boy or girl who acts like the opposite gender (76.5% vs. 67.7%, p < 0.001).

Turning to comfort with one’s body and emerging sexuality, boys were more likely than girls to report a positive body image (77.1% vs. 65.2%), also to report feeling uncomfortable with pubertal changes (22.4% vs. 13.4%, p < 0.001). Feelings of guilt related to sexuality, such as touching one’s own private parts, having sexual feelings, and/or feeling romantically attracted to someone else, were very common overall, and more so among girls (89.1% vs. 76.3% among boys, p < 0.001). Notably, almost one in five participants (17.5%) reported not knowing whether it is normal for adolescents to be curious about love and sexuality, although agreement was higher for boys than girls (59.3% vs. 49.7%, p < 0.001).

Sex differences were also apparent in terms of relational self-efficacy, with boys being more likely than girls to report high self-efficacy to communicate romantic feelings (49.3% vs. 32.8%) and prevent pregnancy (17.8% vs. 8.5%), whereas self-efficacy to say no to unwanted (sexual) interactions was higher for girls (62.5% vs. 50.5%) (p < 0.001).

Finally, while half (50.0%) of participants reported never experiencing peer bullying, boys more commonly reported being bullied by other boys or girls (47.4% vs. 33.4%), and girls by boys only (14.3% vs. 5.3% of boys being bullied only by girls) (p < 0.001). Among those who had experienced bullying (N = 2155), 40.9% reported that this was due to them being a boy vs. girl, or because they acted in a gender non-confirmatory way. Girls were more likely to report never experiencing physical violence (90.5% vs. 75.1%, p < 0.001), and boys were more likely to report being victims of physical violence irrespective of the perpetrator’s sex (18.5% vs. 5.9% had been victimized by both boys and girls, and 6.4% vs. 3.6% by the opposite sex only).

### Multivariable analyses of selected sexual wellbeing indicators

Correlations between the five selected sexual wellbeing indicators are shown in Table 4. The *rho* coefficients of several indicators were low (under ± 0.15) signaling that these are related, yet distinct, constructs of sexual wellbeing.

**Table 4:**
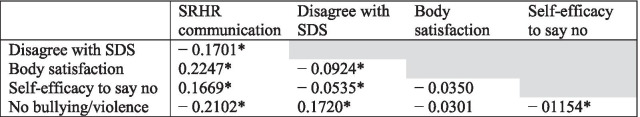
Correlations between five different indicators for sexual wellbeing (*rho*)

*p < 0.05, **p < 0.01, ***p < 0.001

Results from the multivariable regression models (Tables 5 and 6) highlighted differential patterns associated with each sexual wellbeing indicator for boys vs. girls. Due to space constraints and the large number of outcomes explored, only findings from the multivariable analysis are presented here; results from the bivariate analyses including crude ORs are shown in Additional file 1: Appendix Tables 2 and 3. We only retained covariates that contributed to each of the different model using goodness-of-fit statistics, and the grey areas in Tables 5 and 6 therefore show covariates that were not included in the adjusted model for that specific outcome and sex.

**Table 5:**
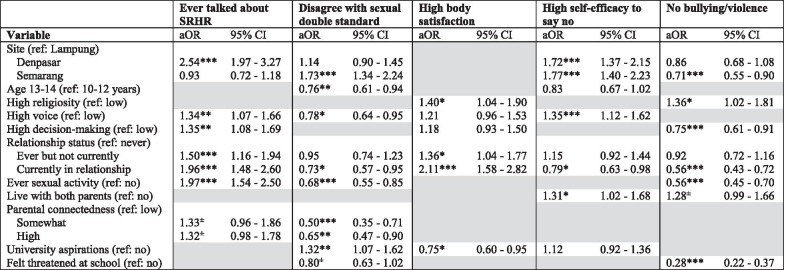
Multivariable analysis of selected sexual wellbeing indicators: boys (N = 2031)

***p < 0.001, **p < 0.01, *p < 0.05, ^±^p < 0.10

**Table 6:**
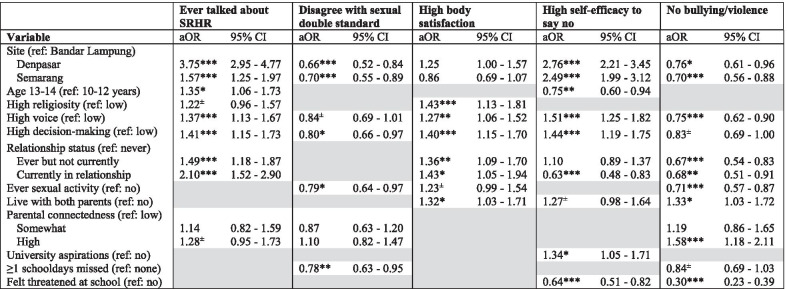
Multivariable analysis of selected sexual wellbeing indicators: girls (N = 2278)

***p < 0.001, **p < 0.01, *p < 0.05, ^±^p < 0.10

### Boys

For boys, those living in Denpasar relative to Bandar Lampung, and those who reported higher voice and decision-making, as well as past/current romantic relationship or sexual activity were more likely to report *ever talking to someone about SRHR.* Meanwhile, boys living in Semarang and boys with greater educational aspirations were more likely to *reject sexual double standard norms*; the same variable was also negatively associated with older age, high parental connectedness, greater perceived voice, being in a romantic relationship, and lifetime experience of sexual activities. In contrast, the likelihood of reporting *positive body satisfaction* was greater for boys with high religiosity, romantic relationship experience, and lower for boys with greater educational aspirations. Boys were more likely to perceive high *self-efficacy to reject unwanted (sexual) advances* if they lived in Denpasar or Semarang relative to Bandar Lampung, lived with both parents, and reported higher perceived voice; but less likely to do so if they were currently in a romantic relationship. *Freedom from peer violence or bullying* among boys was positively associated with high religiosity, and negatively linked with feeling threatened at school, greater perceived decision-making, currently being in a romantic relationship, and lifetime experience of sexual activities (Table 5).

### Girls

For girls, those living in Denpasar or Semarang relative to Bandar Lampung were more likely to have *talked with others about SRHR*; the same variable was positively associated with: older age, greater perceived voice and decision-making, and romantic relationship experiences. Similarly, girls who lived in Denpasar or Semarang, with greater voice and decision-making were more likely to perceive greater *self-efficacy to say no*; this outcome was also positively associated with higher educational aspirations, and negatively linked with older age, feeling threatened at school and being in a relationship. *Positive body satisfaction* was associated with high voice and decision-making, as well as with high religiosity, romantic relationship experience, and living with both parents. In contrast, *rejecting the sexual double standard* was less common among girls who reported greater decision-making, those living in Denpasar or Semarang, those who had missed schooldays, and who reported ever engaging in a sexual activity. Similarly, *freedom from peer bullying and violence* among girls varied by site, and was less likely among those with greater perceived voice, those with experience of past/current romantic relationships, or sexual activities, and those who reported feeling threatened at school. Whereas, living with both parents and high parental connectedness increased the likelihood of reporting no experiences of bullying or violence among girls (Table 6).

## Discussion

This study adds to the small but growing body of evidence exploring positive aspects of adolescent sexuality. It is one of the first to measure sexual wellbeing from a broad perspective among 10–14-year-olds, especially in an Asian context where sexual norms remain restrictive [23]. Our findings suggest that while boys and girls in these Indonesian settings score high on some aspects of sexual wellbeing (such as body satisfaction), unequal gender attitudes, misconceptions, guilt and uncertainties related to sexuality are common, and self-efficacy to prevent pregnancy is very low. The high levels of sexual guilt and misperceptions confirm the low SRHR knowledge found in the 2017 national DHS study with older youth (ages 15–24-years), where few had received any school-based information on family planning [16]. Indeed, previous qualitative studies show how Indonesian parents and young people alike avoid talking about puberty and sexuality due to the perceived irrelevance and taboo of the topics [23, 24].

While comparing our findings to global research is challenging due to the lack of validated measures and inclusion of 10–14-year-olds, it aligns with how a 2018 Ugandan study found that most young adolescents held a positive body image, but endorsement of unequal gender norms was common [14]. The authors also observed differential patterns for boys and girls—confirming our findings that gender differences in healthy sexuality development appear to manifest early in adolescence.

Specifically, we found that girls reported lower SRHR knowledge, poorer body satisfaction, more feelings of guilt related to sexuality development, and lower self-efficacy to prevent pregnancy than boys. Voice and decision-making seemed to be especially linked with sexual wellbeing for girls, similar to how research with 10–14-year-olds in other urban poor settings have found perceived agency to correlate with SRHR communication [25]. In contrast, boys were more likely to hold gender stereotypical attitudes, less self-efficacy to ‘say no’, and to report having experienced bullying and violence, while simultaneously reporting lower educational aspirations and parental connectedness—both of which were associated with sexual wellbeing.

These differences are likely related to the Indonesian social norms around sexuality and gender that are informed by moral values that considers ‘free’ (pre-marital) sex as ‘dangerous’ and ‘immoral’ [23], and fear that unregulated boy-girl contact might encourage this [26]. Indeed, qualitative data collected by our team in the same study sites show how parents and young people alike avoid talking about puberty and sexuality due to the perceived irrelevance and taboo of the topics [27]. Nonetheless, the fact that girls and boys reported relatively high comfort with their bodies and puberty, but simultaneously expressed high guilt in relation to sexuality development, illustrates the importance of considering adolescent sexual wellbeing as a multi-dimensional construct—moving beyond looking (only) at sexual risk behaviors as is typically done in survey research [5, 8, 14]. It also confirms the call from global key stakeholders such as the UNFPA, UNESCO and WHO, that young people need comprehensive sexuality education on a broad range of topics (including those that might be culturally sensitive) and to apply a positive and holistic focus on well-being [28, 29].

Social norms appears in Indonesia to especially target girls to conform to conventional moral behavior, with boys permitted far greater freedom [30]. Similarly, qualitative findings from six other GEAS sites illustrates gender norms expecting boys to be tough and not to show emotions, while perceiving girls at greater SRHR risk and in need of protection [31]. However, qualitative research with older Indonesian youth highlights how young men face contesting masculinity norms to both become reliable citizens and family providers, while simultaneously living up to ideals of hegemony and risk-taking [32]. Future GEAS waves that follow adolescents as they age in Indonesia and other urban poor sites can help elucidate if gender differences persist, and, if so, whether and how they impact (sexual) health and wellbeing.

We also found that romantic relationship involvement was positively linked with both greater SRHR communication and body satisfaction. Although the specific meaning of these ‘relationships’ is unclear, evidence suggests that they are more social than sexual in nature [33]. In light of findings from previous studies [34], one could hypothesize that having a girlfriend or boyfriend is experienced as a form of ‘social confirmation,’ thereby boosting body satisfaction. Being in a relationship might also provide a context for communicating about feelings and puberty, which were the most commonly discussed topics among both boys and girls. In contrast, romantic relationship experiences were associated with less ability to reject unwanted (sexual) advances as well as more peer violence victimization. This is somewhat expected given the young age and context, where early dating or involvement in sexual activities might be(come) unwanted [9].

Finally, we found that sexual wellbeing varied by study site, highlighting the role of local contexts in shaping healthy developmental trajectories. These differences likely reflect the cultural variability of the sample across Bandar Lampung, Semarang and Denpasar in terms of religion and level of modernization, which in turn represent the Indonesian multi-cultural society. Denpasar (Bali) is mostly dominated by Hindu religion and is in addition a well-known tourist destination, and thus more exposed to internationalization and influences from different cultures, including those more ‘Western’. The level of modernization in Bandar Lampung is considered less advanced compared to Denpasar, while Semarang (Central Java) is in between. In terms of religion, Bandar Lampung is dominated by Islam and it is generally more conservative than Semarang [18]. This may help explain why both boys and girls from Bandar Lampung generally reported lower sexual wellbeing than those in Denpasar, and sometimes Semarang, based on the indicators included in our study (e.g. less likely to ever have talked about SRHR with someone else, or to express self-efficacy to reject unwanted advances). The conservative nature of Bandar Lampung is also reflected in the lower response rate in this study site, where some students felt uncomfortable participating in the GEAS survey, or were not allowed to do so by their parents, due to its sensitive nature (e.g., including questions about kissing, contraception, oral sex and sexual intercourse) [18]. The vast differences between these study sites highlight the importance of not generalizing findings across Indonesia, and to stratify future findings by site. For this reason, we have also developed site-specific reports in Bahasa-Indonesia (local language) as part of the broader Explore4Action project in order to guide regional decision-makers, health and educational practitioners.

## Limitations

There are a number of limitations to our study, including the cross-sectional baseline data which precludes conclusions about causality. The longitudinal design will, however, allow for further exploration of associations over time. The high levels of missing responses may constitute another limitation; we addressed this issue via sensitivity analyses. Given the minimal differences between the full and complete case data sets, we feel that the use of the large sample benefitted the analysis. While we could have used the complete case dataset, this type of listwise deletion is problematic when the reasons for missingness may not be random, which may be the case in surveys on sensitive topics like adolescent sexuality. Such an approach would have excluded much of the participant’s data from the current analysis, which may create bias and greatly reduces the power of the dataset [35]. As in any study about adolescent sexuality, the survey might also have been subject to social desirability bias; we addressed this risk by emphasizing the privacy and voluntary nature of the study. Finally, because the study focused on adolescents in selected schools from three different urban sites, findings are not generalizable within or beyond the Indonesian context. Nonetheless, to our knowledge this is the first large-scale quantitative study to assess healthy adolescent sexuality rather than sexual risk using a variety of validated measures in a setting where this topic remains understudied.

## Conclusions

Our findings call for public health interventions that target adolescents, as well as their broader social contexts (including parents, teachers and community members) and existing policies to address restrictive and discriminatory norms related to gender, power and sexuality [3]. And while multi-component, multi-level interventions are needed to fully address underlying social norms [36, 37], interventions that target adolescents can be implemented while simultaneously advocating for broader structural changes and working with parents and communities [38]. In particular, comprehensive sexuality education (CSE) can play an important role to support adolescent sexual wellbeing by providing evidence-based, contextually and developmentally-appropriate information across different time points [3]. Global evidence shows that when implemented well, CSE can successfully improve adolescent SRHR [13, 39–42], bolstering knowledge, self-efficacy, communication and decision-making skills [13, 40, 41]. Such education should start early, follow the UNESCO international technical guidance for CSE [28], be delivered by motivated, trained educators (such as teachers or health professionals who have been trained to deliver CSE), and not only aim at prevention of risk behavior, but to support young people’s positive understanding of their own development and build skills to navigate their complex and sometimes contradictory social realities [13, 28, 41]. School-based CSE that addresses gender and power dynamics including harmful masculinity norms [42] may also provide a tool to reduce the relatively high levels of peer bullying and violence found in the current study, especially for boys—which, unless addressed, might manifest as intimate partner violence later in adolescence [43, 44].

In terms of research implications, our results confirm both the importance and complexity of measuring sexual wellbeing in adolescence, being among the first to explore this among the youngest age groups in a low-income country. There is always a desire for simple solutions based on linear relationships between factors, but the present research shows that data rarely align with such simplicity. For example, high decision-making among boys was associated with more SRHR communication, but inversely associated with freedom from violence. Consistent with previous research, greater agency, for example, does not appear to be equally associated with all positive outcomes [22] (e.g. reduced interpersonal violence). Rather, our results confirm sexual wellbeing as a multi-dimensional phenomenon, calling for studies to move beyond traditional outcomes such as unintended pregnancy and HIV/STI to include a broader range of validated measures. Future longitudinal studies will be critical to better understand healthy adolescent sexuality development and could provide more specific direction for designing and evaluating CSE and other adolescent SRHR interventions. We encourage researchers in adolescent SRHR to continue exploring sexual wellbeing across the life-course with representative samples across different cultural settings.

## Supplementary Information

**Additional file 1: Appendix Table 1.** Measures and operationalization of covariates. **Appendix Table 2.** Bivariate analysis of selected sexual wellbeing indicators: Boys (N = 2031). **Appendix Table 3.** Bivariate analysis of selected sexual wellbeing indicators: Girls (N = 2278).

## Data Availability

The datasets used and/or analysed during the current study are available from the corresponding author on reasonable request.

## Abbreviations

SRHR: Sexual and Reproductive Health and Rights
GEAS: Global Early Adolescent Study
SDS: Sexual Double Standard
GST: Gender Stereotypical Traits
GSR: Gender Stereotypical Roles
kNN: K-nearest Neighbor
AIC: Akaike’s Information Criteria
DHS: Demographic and Health Survey
CSE: Comprehensive Sexuality Education
HIV: Human Immunodeficiency Virus
STI: Sexually Transmitted Infections
