# Supplementary material for: Sexual wellbeing in early adolescence: a cross-sectional assessment among girls and boys in urban Indonesia
Source: Reprod Health. 2021 Jul 20;18:153. doi: 10.1186/s12978-021-01199-4 (PMC8290574; doi:10.1186/s12978-021-01199-4)
Supplement: Supplementary file 1 — Additional file 1: Appendix Table 1. Measures and operationalization of covariates. Appendix Table 2. Bivariate analysis of selected sexual wellbeing indicators: Boys (N = 2031). Appendix Table 3. Bivariate analysis of selected sexual wellbeing indicators: Girls (N = 2278). [file 12978_2021_1199_MOESM1_ESM.docx]

**Appendix Table 1.** Measures and operationalization of covariates

| **Variable** | **Survey question(s) and response options** | **Measurement in analysis** |
| --- | --- | --- |
| Study site | (Automatically generated via sampling) | 1=Bandar Lampung  2=Denpasar  3=Lampung |
| Age | How old are you? (IA1) | Continuous  Dichotomous  0=10-12  1=13-14 |
| Started puberty | GIRLS: Have you started to have periods? (VIIIB2)  BOYS: Have you started puberty? For example, have your penis or testicles (balls) started to get larger compared to when you were younger? (VIIIC1)  (Yes, No, Don’t know) | Dichotomous  0=No/Don’t know  1=Yes |
| Sex | Are you a…?  (Boy, Girl) | Dichotomous  0=Boy  1=Girl |
| Religion | What is your religion? (IA6religion_in) | Categorical  0=Islam  1=Hinduism  2=Christian/other |
| Religiosity | How important is religion to you? (IA7)  (1=not important at all, 2=not very important, 3=somewhat important, 4=very important) | Dichotomous  0=low (1-2)  1=high (3-4) |
| Perceived decision making | How often are you able to make the following decisions on your own without an adult?   - What clothes to wear when you are not in school/working (XIC1A) - What to do in your free time (XIC1B) - What to eat when you are not at home (XIC1C) - Who you can have as friends (XIC1E)   (4=often, 3=sometimes, 2=rarely, 1=never) | Continuous 4-item scale, composite (mean) score 1-4  Dichotomous  0=median or below  1= above median |
| Perceived voice | *How often are the following statements true for you?*   - My parents or guardians ask for my opinions on things (XIB1a) - My parents or guardians listen when I share my opinions (XIB1b) - My friends ask my advice when they have a problem (XIB1c) - If I see something wrong in school or the neighborhood I feel I can tell someone and they will listen (XIB1d) - I can speak up in class when I have a comment or question I can speak up when I see someone else being hurt (XIB1e) - I can ask adults for help when I need it (XIB1f)   (4=often, 3=sometimes, 2=rarely, 1=never) | Continuous 8-item scale, composite (mean) score 1-4  Dichotomous  0=median or below  1= above median |
| Relationship status | *Today, which statement best describes you?* (XA11)  (married, engaged to be married to someone, have a boy/girlfriend, have more than one boy/girlfriend, not currently in a relationship but I had one in the past, I have never been in a romantic relationship, other, don’t know) | 0=never in relationship  1= ever but not currently  2= currently in relationship (married, engaged, boy/girlfriend) |
| Ever non-coital sex (held hands, hugged, kissed, touched) | *Now we would like to ask you about things that YOU might have done together with someone else as more than just friends (i.e. with a boyfriend/girlfriend). Remember that you can skip any question that you do not feel comfortable answering. You should also indicate if you don’t understand the question.*   - Have you ever spent time alone with someone you were in love with in a private space without any adults around? (XC4) - Have you ever held hands with someone you were in love with? (XC5) - Have you ever hugged or cuddled with someone you were in love with? (XC6) - Have you ever kissed or been kissed by someone on the lips or with your tongue? (XC7) - Have you ever touched another boy or girls’ private parts or been touched by someone? (XC10)   (Yes, No, Don’t know) | 0=no (no or don’t know to all)  1=yes (yes to any of the options) |
| Ever sexual intercourse | - Have you ever had sexual intercourse? *(By this we mean when a boy or man puts his penis in a girl’s or woman’s vagina.)*  (XC11)   (Yes, No, Don’t know) | 0=no/don’t know  1=yes |
| Main caregiver | Who is the person who most looks after you/takes care of you? This is sometimes called your primary or main caregiver. (IIA1)  (mother, step-mother, father, step-father, brother, sister, grandmother, grandfather, aunt, uncle, other adult family member, other adult non-family member, other, there is no one that takes care of me) | Categorical  0=mother  1=father  2=sibling  3=grandparent  4=other adult family member  5=other |
| Living with | Who are living with you? (IIA5)  (mother/step-mother, father/step-father, older sister, younger sister, older brother, younger brother, grandmother/father, aunt/uncle, cousin, other people from the community) | 0=both parents  1=mom/dad only  2=grandparents/other |
| Parental connctedness | Do you feel close to your main caregiver? (By close, we mean that you can talk to that person and tell them about personal and important things). (IIB4)  1=not at all, 2=not much, 3=somewhat, 4=a lot) | Categorical  0=low (1-2)  1=somewhat (3)  2=high (4) |
| School aspiration | How much school do you think you will complete? (IVA2_ja) | 0=junior high or below  1=senior high or diploma  2=university  3=other |
| Number of school days missed last month | During the past month, how many days did you miss school for any reason except when school was closed or for holidays? (IVA7) | Categorical  0=none  1=1-2  2=3-4  3=5 or more |
| Felt unsafe at school | Can you tell me where you felt unsafe or threatened?   - Responded: ”in your classroom” (VA5_school) | 0=no  1=yes |
| Social media use | For each item, please tell me if you have access to it…   - Responded: “Social media account such as Twitter, Instagram, Whatsapp or Facebook” (VIA1E) | 0=no  1=yes |

**Appendix Table 2.** Bivariate analysis of selected sexual wellbeing indicators: Boys (N=2031)

|  | **Ever talked about SRH** | **Disagree with sexual double standard** | **Positive body image** | **High self-efficacy to say no** | **Freedom from bullying and violence** |
| --- | --- | --- | --- | --- | --- |
| **Variable** | **OR (95% CI)** | **OR (95% CI)** | **OR (95% CI)** | **OR (95% CI)** | **OR (95% CI)** |
| Site |  |  |  |  |  |
| Lampung | 1.00 | 1.00 | 1.00 | 1.00 | 1.00 |
| Denpasar | 2.31 (1.81, 2.95)*** | 1.21 (0.96, 1.52) | 1.01 (0.78, 1.31) | 1.86 (1.49, 2.32)*** | 0.94 (0.76, 1.17) |
| Semarang | 0.89 (0.71, 1.13) | 1.73 (1.35, 2.22)*** | 1.03 (0.79, 1.35) | 1.87 (1.48, 2.35)*** | 0.78 (0.62, 0.98)* |
| Age |  |  |  |  |  |
| 10-12 years | 1.00 | 1.00 | 1.00 | 1.00 | 1.00 |
| 13-14 years | 1.06 (0.85, 1.31) | 0.71 (0.58, 0.88)** | 1.06 (0.84, 1.34) | 0.76 (0.63, 0.93)** | 0.96 (0.79, 1.17) |
| Religiosity |  |  |  |  |  |
| Low | 1.00 | 1.00 | 1.00 | 1.00 | 1.00 |
| High | 1.04 (0.78, 1.38) | 1.15 (0.86, 1.52) | 1.35 (1.00, 1.82)**^±^** | 0.90 (0.69, 1.17) | 1.38 (1.05, 1.81)* |
| Perceived voice |  |  |  |  |  |
| Low | 1.00 | 1.00 | 1.00 | 1.00 | 1.00 |
| High | 1.51 (1.24, 1.85)*** | 0.79 (0.65, 0.96)* | 1.28 (1.03, 1.60)* | 1.37 (1.14, 1.64)** | 0.91 (0.76, 1.09) |
| Perceived decision-making |  |  |  |  |  |
| Low | 1.00 | 1.00 | 1.00 | 1.00 | 1.00 |
| High | 1.51 (1.23, 1.86)*** | 0.82 (0.67, 1.00)* | 1.26 (1.00, 1.58)* | 1.04 (0.87, 1.26) | 0.74 (0.61, 0.89)* |
| Relationship status |  |  |  |  |  |
| Never in relationship | 1.00 | 1.00 | 1.00 | 1.00 | 1.00 |
| Ever but not currently | 1.63 (1.28, 2.08)*** | 0.85 (0.67, 1.08) | 1.35 (1.04, 1.75)*** | 1.17 (0.94, 1.46) | 0.70 (0.56, 0.88)*** |
| Currently in relationship | 2.43 (1.88, 3.14)*** | 0.55 (0.44, 0.70)*** | 2.18 (1.63, 2.91)*** | 0.71 (0.58, 0.89)** | 0.44 (0.35, 0.55)*** |
| Ever sexual activity |  |  |  |  |  |
| No | 1.00 | 1.00 | 1.00 | 1.00 | 1.00 |
| Yes | 2.30 (1.84, 2.87)*** | 0.59 (0.48, 0.71)*** | 1.40 (1.11, 1.76)** | 0.98 (0.81, 1.18) | 0.45 (0.37, 0.55)*** |
| Living with both parents |  |  |  |  |  |
| No | 1.00 | 1.00 | 1.00 | 1.00 | 1.00 |
| Yes | 0.98 (0.76, 1.28) | 1.06 (0.82, 1.38) | 1.00 (0.75, 1.33) | 1.43 (1.12, 1.82)** | 1.33 (1.04, 1.71)* |
| Parental connectedness |  |  |  |  |  |
| Low | 1.00 | 1.00 | 1.00 | 1.00 | 1.00 |
| Somewhat | 1.40 (1.02, 1.92)** | 0.53 (0.37, 0.75)** | 1.19 (0.85, 1.67) | 1.25 (0.93, 1.69) | 0.89 (0.66, 1.20) |
| High | 1.48 (1.12, 1.96)** | 0.62 (0.45, 0.86)** | 1.44 (1.06, 1.95)* | 1.231(1.0, 1.72) | 1.00 (0.77, 1.32) |
| University aspirations |  |  |  |  |  |
| No | 1.00 | 1.00 | 1.00 | 1.00 | 1.00 |
| Yes | 1.01 (0.83, 1.24) | 1.42 (1.16, 1.73)** | 0.73 (0.58, 0.92)** | 1.27 (1.05, 1.53)* | 1.15 (0.95, 1.39) |
| Schooldays missed last month |  |  |  |  |  |
| None | 1.00 | 1.00 | 1.00 | 1.00 | 1.00 |
| ≥1 schooldays | 1.29 (1.05, 1.58)* | 0.85 (0.69, 1.03) | 1.19 (0.95, 1.48) | 1.14 (0.95, 1.37) | 0.88 (0.73, 1.06) |
| Ever felt threatened at school |  |  |  |  |  |
| No | 1.00 | 1.00 | 1.00 | 1.00 | 1.00 |
| Yes | 1.14 (0.90, 1.45) | 0.78 (0.62, 0.98)* | 0.98 (0.76, 1.27) | 1.11 (0.89, 1.37) | 0.28 (0.21, 0.36)*** |

***p<0.001, **p<0.01, *p<0.05, ±p<0.10

**Appendix Table 3.** Bivariate analysis of selected sexual wellbeing indicators: Girls (N=2278)

|  | **Ever talked about SRH** | **Disagree with sexual double standard** | **Positive body image** | **High self-efficacy to say no** | **Freedom from bullying and violence** |
| --- | --- | --- | --- | --- | --- |
| **Variable** | OR (95% CI) | OR (95% CI) | OR (95% CI) | OR (95% CI) | OR (95% CI) |
| Site |  |  |  |  |  |
| Lampung | 1.00 | 1.00 | 1.00 | 1.00 | 1.00 |
| Denpasar | 3.94 (3.12, 4.98)*** | 0.62 (0.49, 0.79)*** | 1.38 (1.10, 1.72)*** | 2.85 (2.29, 3.54)*** | 0.71 (0.57, 0.87)*** |
| Semarang | 1.70 (1.37, 2.11)*** | 0.66 (0.52, 0.83)*** | 0.93 (0.75, 1.15) | 2.43 (1.96, 3.02)*** | 0.60 (0.48, 0.74)*** |
| Age |  |  |  |  |  |
| 10-12 years | 1.00 | 1.00 | 1.00 | 1.00 | 1.00 |
| 13-14 years | 1.40 (1.11, 1.78)** | 0.86 (0.69, 1.08) | 1.07 (0.86, 1.34) | 0.76 (0.62, 0.95)* | 0.94 (0.76, 1.17) |
| Religiosity |  |  |  |  |  |
| Low | 1.00 | 1.00 | 1.00 | 1.00 | 1.00 |
| High | 1.45 (1.15, 1.82)** | 1.02 (0.80, 1.29) | 1.48 (1.18, 1.85)** | 1.24 (0.99, 1.56) | 1.24 (0.99, 1.55) |
| Perceived voice |  |  |  |  |  |
| Low | 1.00 | 1.00 | 1.00 | 1.00 | 1.00 |
| High | 1.70 (1.43, 2.04)*** | 0.78 (0.66, 0.94)** | 1.43 (1.20, 1.70)*** | 1.77 (1.49, 2.10)*** | 0.73 (0.62, 0.86)*** |
| Perceived decision-making |  |  |  |  |  |
| Low | 1.00 | 1.00 | 1.00 | 1.00 | 1.00 |
| High | 1.54 (1.27, 1.86)*** | 0.76 (0.63, 0.91)** | 1.49 (1.24, 1.79)*** | 1.58 (1.32, 1.89)*** | 0.75 (0.63, 0.89)** |
| Relationship status |  |  |  |  |  |
| Never in relationship | 1.00 | 1.00 | 1.00 | 1.00 | 1.00 |
| Ever but not currently | 1.49 (1.20, 1.85)*** | 0.78 (0.64, 0.96) | 1.34 (1.09, 1.66)** | 1.20 (0.97, 1.47) | 0.56 (0.46, 0.68)*** |
| Currently in relationship | 1.98 (1.46, 2.70)*** | 0.86 (0.65, 1.14) | 1.49 (1.12, 1.99)** | 0.69 (0.53, 0.89)** | 0.54 (0.41, 0.70)*** |
| Ever sexual activity |  |  |  |  |  |
| No | 1.00 | 1.00 | 1.00 | 1.00 | 1.00 |
| Yes | 1.19 (0.97, 1.47) | 0.76 (0.62, 0.93)** | 1.34 (1.09, 1.65)** | 0.86 (0.71, 1.04)* | 0.55 (0.46, 0.67)*** |
| Living with both parents |  |  |  |  |  |
| No | 1.00 | 1.00 | 1.00 | 1.00 | 1.00 |
| Yes | 1.22 (0.95, 1.58) | 1.00 (0.77, 1.30) | 1.30 (1.01, 1.66)* | 1.26 (0.99, 1.61) | 1.37 (1.08, 1.75)* |
| Parental connectedness |  |  |  |  |  |
| Low | 1.00 | 1.00 | 1.00 | 1.00 | 1.00 |
| Somewhat | 1.20 (0.88, 1.65) | 0.87 (0.63, 1.20) | 1.09 (0.80, 1.49) | 1.28 (0.94, 1.74) | 1.38 (1.01, 1.87)*** |
| High | 1.29 (0.98, 1.70) | 1.11 (0.83, 1.48) | 1.32 (1.01, 1.74)* | 1.24 (0.95, 1.63) | 1.87 (1.42, 2.45)*** |
| University aspirations |  |  |  |  |  |
| No | 1.00 | 1.00 | 1.00 | 1.00 | 1.00 |
| Yes | 1.24 (0.98, 1.58) | 1.13 (0.89, 1.44) | 1.02 (0.80, 1.29) | 1.52 (1.21, 1.91)*** | 1.05 (0.84, 1.32) |
| Schooldays missed last month |  |  |  |  |  |
| None | 1.00 | 1.00 | 1.00 | 1.00 | 1.00 |
| ≥1 schooldays | 1.19 (0.97, 1.45) | 0.76 (0.62, 0.92)** | 1.01 (0.83, 1.22) | 1.04 (0.86, 1.26) | 0.81 (0.68, 0.98)* |
| Ever felt threatened at school |  |  |  |  |  |
| No | 1.00 | 1.00 | 1.00 | 1.00 | 1.00 |
| Yes | 1.09 (0.85, 1.39) | 0.82 (0.64, 1.04) | 1.09 (0.85, 1.39) | 0.69 (0.54, 0.86)*** | 0.29 (0.22, 0.37)*** |
